# Bovine Muscle Satellite Cell-Derived Exosomes Modulate Preadipocyte Adipogenesis via bta-miR-2904

**DOI:** 10.3390/ani16020218

**Published:** 2026-01-12

**Authors:** Mengxia Sun, Mengdi Chen, Yang Yi, Binru Li, Tianyu Zhang, Ziqi Liu, Wenyu Jiao, Tianqi Si, Yunkai He, Guangjun Xia

**Affiliations:** Department of Animal Science, College of Agriculture, Yanbian University, Yanji 133000, China; smengxia11160928@163.com (M.S.);

**Keywords:** co-culture, exosome, bta-miR-2904, adipogenic differentiation, cattle

## Abstract

Intramuscular fat (IMF) significantly impacts meat quality. Exosomes have attracted increasing attention for their regulatory roles in muscle-adipose tissue crosstalk; however, their precise mechanisms remain largely unclear. Based on this, this study aimed to establish a muscle-adipose co-culture system to better simulate the in vivo physiological environment. Using exosomal miRNAs as molecular links, we investigated how bovine muscle satellite cells influence lipid accumulation and adipogenesis in preadipocytes. We established a co-culture system of bovine muscle satellite cells and preadipocytes and found that co-culture significantly inhibited lipid droplet accumulation and adipogenesis in preadipocytes. Therefore, we hypothesized that exosomes derived from bovine muscle satellite cells regulate the adipogenic differentiation of bovine preadipocytes through intercellular communication and that specific exosomal miRNAs play pivotal roles in this regulatory process. We successfully isolated and identified muscle-derived (Mu-EXO), adipose-derived (Ad-EXO), and co-culture exosomes (Co-EXO). High-throughput sequencing revealed the differential expression profiles of miRNAs. Notably, the bovine-specific miRNA bta-miR-2904, annotated in miRBase v22 with limited cross-species conservation, was significantly enriched in Mu-EXO and Co-EXO compared with Ad-EXO. Further functional experiments demonstrated that overexpression of bta-miR-2904 markedly inhibited lipid droplet accumulation, triglyceride content, and the expression of adipogenesis-related genes in preadipocytes; inhibition had opposite effects. Our results demonstrate that bovine muscle-derived exosomal miR-2904 inhibits lipid accumulation and adipogenesis in preadipocytes. These results establish a theoretical basis for understanding skeletal muscle-adipose crosstalk and offer a novel molecular target for regulating intramuscular fat deposition in beef cattle.

## Simple Summary

Intramuscular fat, commonly referred to as marbling, plays a crucial role in determining meat tenderness, juiciness, and flavor. In this study, we established a co-culture system comprising bovine muscle satellite cells and preadipocytes to simulate the in vivo microenvironment and elucidate the mechanism by which exosomal miRNAs regulate adipogenesis. Our experiments demonstrated that the bovine-specific miRNA bta-miR-2904 significantly inhibits lipid droplet accumulation and downregulates the expression of key adipogenesis-related genes in preadipocytes. These findings not only offer new insights into muscle–fat interactions but also highlight bta-miR-2904 as a potential molecular target for regulating intramuscular fat deposition in beef cattle.

## 1. Introduction

Intramuscular fat (IMF), primarily deposited between muscle bundles and fibers, serves as the core material basis for the marbling characteristics of meat. Its content and spatial distribution are pivotal determinants of sensory meat quality, including tenderness, color, and juiciness [1]. While nutritional regulation strategies can augment intramuscular fat (IMF) content, their efficacy is often constrained. These interventions primarily influence overall fat deposition and frequently result in excessive accumulation of subcutaneous and visceral fat, consequently elevating the risk of metabolic disorders in animals [2,3]. Furthermore, excessive IMF can impair muscle function [4]. Therefore, elucidating the molecular mechanisms governing IMF deposition and its precise regulation through genetic or molecular biological approaches is crucial for optimizing meat quality and safeguarding animal health.

Acting as key endocrine organs, skeletal muscle and adipose tissue establish elaborate bidirectional crosstalk through the elaboration of cytokines and exosomes. For instance, during muscle injury repair, exosomal miR-127-3p derived from fibro/adipogenic progenitors (FAPs) promotes the activation, proliferation and differentiation of muscle satellite cells (MuSCs) by targeting and degrading Sphingosine-1-phosphate receptor 3 (*S1pr3*). Conversely, MuSCs and their descendant myoblasts/myotubes deliver miR-206-3p and miR-27a/b-3p via exosomes to effectively inhibit lipid accumulation and adipogenic differentiation in FAPs by targeting Peroxisome proliferator-activated receptor gamma (*PPARγ*) and Runt-related transcription factor 1 (*Runx1*), thereby attenuating intramuscular fat infiltration and ensuring sustained muscle regeneration [5]. Various myokines secreted by skeletal muscle have been demonstrated to exert inhibitory effects on adipocytes. For instance, irisin, a prominent myokine, reduces lipid deposition in 3T3-L1 adipocytes by upregulating the expression of key lipolytic genes, such as Adipose triglyceride lipase (*ATGL*), Hormone-sensitive lipase (*HSL*), and Perilipin 1 (*PLIN1*), and regulating the PI3K-AKT pathway [6]. Additionally, myostatin (*MSTN*) diminishes fat accumulation in adipocytes by inhibiting differentiation, regulating fatty acid saturation, and promoting lipolysis [7,8]. Muscle-derived metabolites have also been demonstrated to make a critical contribution in muscle–adipose tissue crosstalk. Metabolomic and transcriptomic analyses of the longissimus dorsi muscle in obese and lean pigs at different developmental stages revealed pronounced differences in skeletal muscle lipid metabolism between the two groups. Notably, metabolites such as fumarate, succinate, L-lysine, 2-hydroxyisovalerate, and L-carnitine showed strong correlations with IMF deposition, suggesting that they may function as intercellular signaling molecules regulating the process of IMF formation [9]. MicroRNAs (miRNAs) are characterized as small, non-coding RNA species, usually ranging from 19 to 25 nucleotides in length. These miRNAs are incorporated into multivesicular bodies (MVBs) and released from the parental cell via the exosomal pathway upon MVB fusion with the plasma membrane [10]. It is estimated that in different animal species, about 30–80% of genes are subject to miRNA regulation [11], highlighting their involvement in an extensive and complex regulatory network that governs cellular development. Exosomes, membrane-bound vesicles ranging from 40 to 200 nm in diameter [12], function as pivotal mediators of intercellular communication [13]. They facilitate crosstalk between organs and tissues by transporting a cargo of bioactive molecules, including nucleic acids, proteins, and lipids. Previous studies have demonstrated that exosomal miRNAs account for roughly 70% of the modulatory impacts on recipient cells, followed by proteins, which account for about 10% [14].

In the context of muscle-adipose tissue crosstalk, muscle-derived exosomes and their constituent miRNAs are pivotal regulators of adipogenic differentiation. For instance, high-throughput sequencing has identified 104 distinctly expressed miRNAs between porcine muscle-derived and adipose-derived exosomes, with subsequent studies confirming that muscle-derived exosomes can significantly inhibit the proliferation and differentiation of porcine preadipocytes [15]. Moreover, extracellular vesicles (EVs) from MuSCs significantly inhibit adipocyte viability, reduce lipid droplet accumulation in preadipocytes, and suppress adipogenic differentiation. Among the miRNAs within these EVs, miR-146a-5p is highly abundant and acts as a pivotal regulator of this process [16]. Skeletal muscle-derived miR-146a-5p significantly inhibits adipogenic differentiation and lipid accumulation in preadipocytes by targeting SMAD family member 3 (*Smad3*) for downregulation, which disrupts their maturation program [17]. Furthermore, under cold exposure, murine muscle secretes higher levels of exosomal miR-122 to upregulate mitochondrial uncoupling protein 1 (*UCP1*) and thermogenic genes in adipocytes, thereby promoting browning of subcutaneous fat and reducing fat deposition [18]. Exercise-induced muscle-derived exosomal miR-27a alleviates high-fat diet-induced obesity in mice by enhancing fat browning and activating the IRS1/Akt/GLUT4 signaling pathway [19]. Collectively, these studies illuminate the regulatory functions of muscle-derived exosomes and their miRNAs in adipocyte biology, elucidating the molecular mechanisms of fat-muscle crosstalk. These complex interaction networks reveal the multidimensional regulatory characteristics of fat differentiation, providing a critical theoretical basis for understanding fat deposition.

Although classical cytokines play important roles in the complex network of muscle-adipose crosstalk, the mechanisms of exosome-mediated regulation remain to be fully elucidated. We speculated that bovine MuSCs and preadipocytes engage in intercellular crosstalk and that exosomal miRNAs derived from MuSCs act as key mediators in this communication process. To verify this, we established a co-culture system of bovine muscle satellite cells and preadipocytes. Then we characterized the miRNA expression profiles of muscle-derived exosomes (Mu-EXO), adipose-derived exosomes (Ad-EXO), and co-culture exosomes (Co-EXO), and identified a bovine-specific muscle-derived exosomal miRNA, bta-miR-2904. We further propose that bta-miR-2904 functions as a negative regulator of adipogenic differentiation in preadipocytes. This study elucidates the mechanisms underlying muscle-adipose interactions in vitro and establishes a foundation for subsequent mechanistic studies and in vivo experiments aimed at precisely regulating fat deposition in beef cattle.

## 2. Materials and Methods

### 2.1. Experimental Animals

Preadipocytes and MuSCs were isolated from three 1-week-old Yanbian Yellow cattle calves, provided by Jixing Animal Husbandry Co., Ltd. (Hunchun, China). The animal study protocol was approved by the Medical Ethics Committee of the Affiliated Hospital of Yanbian University (Yanbian Hospital) (Approval number 201702). All experiments were conducted using cells at passage 4 (P4), which were previously cultured and characterized in our laboratory [20].

### 2.2. Cell Isolation

Muscle satellite cells were isolated from the longissimus dorsi muscle of 1-week-old Yanbian yellow cattle calves using the explant adherent culture method. Under aseptic conditions, muscle tissue was collected and immediately placed in pre-cooled phosphate-buffered saline (PBS) containing 10% penicillin–streptomycin (PS Biological Industries, Kibbutz Beit HaEmek, Israel). In a biosafety cabinet, visible fascia, hair, and other debris were carefully removed using sterile ophthalmic scissors and forceps. The tissue was then immersed in 75% ethanol for 10 min for surface disinfection, and washed three times with PBS containing 2% PS. Subsequently, the longissimus dorsi was minced into small explants of approximately 1 mm^3^, rinsed again with PBS containing 2% PS, and evenly distributed at appropriate intervals on the bottom of culture flasks. The flasks were gently inverted, and an appropriate volume of proliferation medium was added to the side without tissue explants. The flasks were then placed in a 37 °C, 5% CO_2_ incubator. After 2 h, the flasks were slowly turned upright so that the medium contacted, but did not completely submerge, the tissue explants. Incubation was continued with medium changes performed every 2 days. When cell migration from the explants reached approximately 70% confluence, the remaining tissue pieces were carefully removed. Once the adherent cells reached about 80% confluence, they were detached with trypsin and seeded into flasks pre-coated with 0.01% poly-L-lysine (Sigma-Aldrich, St. Louis, MO, USA) for purification via differential adhesion. The purified cells were subsequently transferred to regular culture flasks for further expansion and were designated as primary muscle satellite cells.

Preadipocytes were isolated from the adipose tissue of 1-week-old Yanbian yellow cattle calves by enzymatic digestion. Under aseptic conditions, calf adipose tissue was collected and placed in pre-cooled PBS containing 10% PS, ensuring the tissue was fully immersed. In a biosafety cabinet, visible blood vessels and connective tissue were removed using sterile ophthalmic scissors and forceps. The tissue was then immersed in 75% ethanol for 10 min for disinfection and washed three times with PBS containing 2% PS. Afterward, the tissue was minced and repeatedly pipetted with a 1 mL pipette tip until a paste-like consistency was obtained, and the sample was weighed. For digestion, 0.1% collagenase type I was added at a volume ratio of 2:1 (enzyme solution to tissue weight). The tube was gently inverted to fully immerse the tissue. The mixture was incubated at 37 °C in a shaking water bath for 60 min with gentle inversion every 5 min. To terminate the digestion, an equal volume of serum-containing medium was added, followed by centrifugation at 1500× *g* for 10 min, and the supernatant was discarded. The pellet was resuspended in serum-containing medium and sequentially filtered through sterile 100-mesh and 200-mesh filters to remove larger tissue fragments. The filtrate was centrifuged again at 1500× *g* for 10 min, and the supernatant was discarded. The final pellet was resuspended in growth medium and seeded into 25 cm^2^ culture flasks, which were then incubated at 37 °C with 5% CO_2_. The medium was replaced after 24 h of culture and subsequently every 2 days.

### 2.3. Cell Thawing and Culture

Cryopreserved preadipocytes and MuSCs were rapidly thawed from liquid nitrogen in a 37 °C water bath with gentle agitation. The fully thawed cell suspension was transferred to a 15 mL sterile centrifuge tube and centrifuged at 900× *g* for 5 min. The supernatant was discarded, and the cell pellet was resuspended in growth medium. Cells were then counted and seeded at an appropriate density. The growth medium comprised DMEM (Gibco, Grand Island, NY, USA) supplemented with 10% fetal bovine serum (FBS; Biological Industries, Kibbutz Beit HaEmek, Israel) and 2% PS. Cells were maintained at 37 °C in a humidified incubator with 5% CO_2_, and the medium was replaced every 48 h.

### 2.4. Induction of Adipogenic Differentiation

Upon reaching approximately 80% confluence, preadipocytes were induced to differentiate. The growth medium was replaced with induction medium I, containing 0.5 mM IBMX (Sigma-Aldrich, St. Louis, MO, USA), 1 μM dexamethasone (Sigma-Aldrich, St. Louis, MO, USA), and 10 μg/mL insulin (Sigma-Aldrich, St. Louis, MO, USA). After 48 h, the medium was replaced with induction medium II, containing 10 μg/mL insulin. After a further 48 h, the culture medium was exchanged for the standard growth medium, and cells were cultured continuously until full differentiation was achieved.

### 2.5. Immunofluorescence Staining

Isolated muscle satellite cells and preadipocytes were seeded at an appropriate density onto culture dishes containing sterile coverslips. After 12 h of culture, the medium was discarded, and the cells were washed three times with PBS. Cells were then fixed at room temperature with pre-chilled 4% paraformaldehyde for 30 min, followed by three washes with PBS. Permeabilization was performed with 0.1% Triton X-100 for 15 min, and the cells were washed three times with PBS for 5 min each. Subsequently, 5% BSA was added for blocking for 2 h at room temperature. After blocking, the cells were incubated with primary antibodies: Anti-PAX7 (1:100, AB_2843948, Affinity Biosciences, Cincinnati, OH, USA), Anti-PDGF Receptor alpha (1:100, D1E1E, Cell Signaling Technology, Danvers, Massachusetts, USA) at 4 °C overnight. and then with secondary antibody: Goat Anti-Rabbit IgG (H + L) Fluor594-conjugated (1:100, AB_2843436, Affinity Biosciences) at room temperature for 2 h in the dark. The cells were then washed three times with PBST (PBS with 0.1% Tween-20) for 5 min each. Finally, the coverslips were carefully removed from the culture dishes and mounted using Antifade Mounting Medium with DAPI (Beyotime, Shanghai, China). Images were acquired using a fluorescence microscope (Olympus Corporation, Tokyo, Japan).

### 2.6. Co-Culture System

Referring to previous studies [20,21,22], an indirect co-culture model of preadipocytes and MuSCs was established using a Transwell system with a 0.4 μm pore polycarbonate membrane. MuSCs and preadipocytes were seeded into the upper and lower chambers of the Transwell, respectively, at a ratio of 2:1 (MuSCs:preadipocytes).

### 2.7. Oil Red O Staining

After induction of differentiation, preadipocytes were washed three times with PBS and fixed with 1 mL of ice-cold 4% paraformaldehyde at room temperature for 30 min. Following fixation, the cells were washed twice with PBS. The Oil Red O working solution, prepared by mixing stock solution with ddH_2_O at a 3:2 ratio and incubating at room temperature for 20 min, was filtered through a 0.22 μm filter membrane was then added to the cells for 30 min of incubation. After staining, the working solution was discarded, and cells were thoroughly rinsed with ddH_2_O. Staining was evaluated under a light microscope. For quantitative analysis of intracellular lipid droplets, the incorporated Oil Red O was eluted with 60% isopropanol and incubated for 10 min. Absorbance at 510 nm was measured using a multi-functional microplate reader (Spark 10M, Tecan, Männedorf, Switzerland).

### 2.8. Triglyceride Content Determination

Intracellular triglyceride (TG) content was measured using a triglyceride assay kit (Nanjing Jiancheng Bioengineering Institute, Nanjing, China) following the manufacturer’s instructions. Briefly, a lysis buffer was prepared by mixing 1% Triton X-100 (Sigma-Aldrich, USA; preheated at 37 °C for 2 h before dilution) with phenylmethylsulfonyl fluoride (PMSF; Sigma-Aldrich, St. Louis, MO, USA) and phosphatase inhibitor buffer (Sigma-Aldrich, St. Louis, MO, USA) in the recommended proportions. Cells were washed twice with PBS and collected by centrifugation at 900× *g* for 10 min at 4 °C. 300 μL of precooled lysis buffer was added, and cells were lysed on ice for 40 min. The lysates were transferred to a 96-well plate as outlined in Table S1, and the absorbance at 510 nm was measured.

### 2.9. Exosome Isolation and Identification

Exosomes were isolated by ultracentrifugation, with serum concentration gradually reduced to replace the traditional exosome-containing FBS. When the cells reached 80% confluence, the medium was replaced with DMEM containing exosome-depleted FBS (VivaCell, Shanghai, China) and cultured in a 37 °C, 5% CO_2_ incubator. The culture supernatant was collected and centrifuged at 2000× *g* for 30 min at 4 °C to remove cell debris and large particulate impurities, followed by centrifugation at 10,000× *g* for 45 min at 4 °C to separate and remove microvesicles. Subsequently, the resulting supernatant was filtered through a 0.45 μm sterile filter to remove residual organelle fragments and submicron-sized impurities. The filtrate was ultracentrifuged at 100,000× *g* for 70 min at 4 °C. After discarding the supernatant, 10 mL of precooled PBS was added, and a second ultracentrifugation was performed to obtain the exosome pellet. The pellet was then resuspended in 100 μL of precooled PBS, obtaining the exosome suspension for subsequent experiments.

Exosomes were characterized by Transmission Electron Microscopy (TEM) and Nanoparticle Tracking Analysis (NTA). The exosome suspension was dropped onto the surface of a 200-mesh carbon-supported copper grid. After 60 s, excess liquid was removed with filter paper, and 10 μL of 2% uranyl acetate negative stain was immediately dropped onto the center of the copper grid. Following a 60 s incubation, the residual stain was absorbed, and the copper grid was air-dried at room temperature until the stain had completely solidified. The copper grid was then observed under a transmission electron microscope (Hitachi, HT-7700, Tokyo, Japan) to determine the morphology and size of the exosomes. After diluting the exosome suspension was diluted to an appropriate concentration and analyzed using a nanoparticle tracking analyzer (PARTICLE METRIX, ZetaVIEW, Meerbusch, Germany) to determine the particle size distribution.

### 2.10. Exosome Small RNA Sequencing

Exosome small RNA sequencing libraries were constructed using the QIAseq^®^ miRNA Library Kit (Qiagen, Hilden, Germany), strictly following the manufacturer’s instructions. High-throughput sequencing was performed on the Illumina HiSeq 2000 platform (Illumina, Inc., San Diego, CA, USA) with 150 bp paired-end reads.

### 2.11. Cell Transfection

Cells were seeded into 6-well cell culture plates at a density of 1 × 10^6^ cells per well. When cells reached 60% confluence, the medium was replaced with growth medium without PS, and transfection was performed following the Lipofectamine™ 2000 (Thermo Fisher, Waltham, MA, USA) protocol. Briefly, plasmid DNA and Lipo2000 reagent were diluted separately in Opti-MEM, then mixed. The DNA-liposome complexes were incubated at room temperature for 20 min, and then added dropwise to the cell culture plate. Following a 24 h transfection period, the medium was replaced with fresh growth medium. Subsequent experiments were performed after an additional incubation period.

### 2.12. Total RNA Extraction and Real-Time Quantitative PCR

Total RNA was extracted using the Eastep^TM^ Total RNA Extraction Kit (Promega, Shanghai, China). The extracted RNA was reverse transcribed into complementary DNA (cDNA) using the FastKing gDNA Dispelling RT Super Mix kit (TIANGEN, Beijing, China). The RNA reverse transcription system is detailed in Table S2. Quantitative real-time PCR (qRT-PCR) was performed using the SuperReal Pre Mix Plus kit (TIANGEN, Beijing, China), and the reaction system is outlined in Table S3. The PCR program consisted of an initial denaturation at 95 °C for 30 s, followed by 40 cycles of denaturation at 95 °C for 5 s, and annealing at 60 °C for 30 s. All experiments were independently repeated three times. The relative expression of mRNA was calculated using the 2^−ΔΔCT^ method, normalized to GAPDH as the internal reference gene. Specific primers for qRT-PCR were designed using Primer Premier and Oligo 7.0 software, and the primer sequences are listed in Table S4.

### 2.13. Western Blot Analysis

Cell pellets were collected and incubated on ice for 30 min with pre-cooled RIPA lysis buffer (Beyotime, Shanghai, China), containing 1% protease inhibitors. Lysates was then centrifuged at 12,000× *g* for 10 min at 4 °C, and the supernatant, representing total cell protein, was collected. Total protein concentration was determined using an enhanced BCA protein assay kit (Beyotime, Shanghai, China). 20 μg of protein was loaded onto a 10% sodium dodecyl sulfate-polyacrylamide gel electrophoresis (SDS-PAGE) gel (Solarbio, Beijing, China). The gel was transferred to a PVDF membrane (Millipore, Burlington MA, USA) of the same area. The membrane was incubated overnight with primary antibodies: anti-PPARγ (1:1000, AB_2835135, Affinity), Anti-CEBP-alpha (1:1000, A0904, Abclonal, Woburn, MA, USA), anti-GAPDH (1:1000, AB_2107436, Proteintech, Rosemont, IL, USA) at 4 °C, followed by incubation with secondary antibodies: Anti-Rabbit IgG H&L (1:10,000, bs-0295G, Bioss, Erkelenz, Germany), Anti-Mouse IgG H&L (1:10,000, bs-0296R, Bioss) for 1 h at room temperature. Bands were visualized using a chemiluminescence gel imaging analysis system (Alliance MINI HD9, UVITEC, Cambridge, UK), and grayscale values were quantitatively analyzed using ImageJ 1.54 program. GAPDH was used as an internal control.

### 2.14. Bioinformatics Analysis

The raw sequencing data were assessed for quality using FastQC (v0.12.1), and adapter sequences, low-quality reads, and N bases at the ends of sequences were trimmed using fastp (v1.0.1) to yield high-quality clean reads. The NCBI assembly *Bos taurus* assembly (ARS-UCD1.2) and miRBase v22.0 genome were used as references. miRDeep2 was used to identify miRNA expression levels in the samples, and DESeq2 (1.46.0) [23,24] in R (4.4.2) was used for normalization and differential expression analysis. Differentially expressed miRNAs were filtered using a threshold of *p*-value < 0.05 and |log2FC| ≥ 1. Candidate target genes for differentially expressed miRNAs were predicted using miRanda software (v3.3a), with mRNA sequences as references, filtering for results with an Energy-Kcal/Mol < −10 and alignment length (Aln_Len) ≥ 8. Finally, Gene Ontology (GO) [25] and Kyoto Encyclopedia of Genes and Genomes (KEGG) [26] pathway enrichment analyses were conducted on the candidate target genes.

### 2.15. Statistical Analysis

All statistical analyses were performed using SPSS 20.0 software. Data are expressed as mean ± standard error of the mean (SEM), where “n” denotes the number of independent biological replicates. For assays with technical replicates, three technical replicates were performed for each biological replicate. For comparisons between two groups, statistical significance was assessed using an independent two-sample *t*-test. For comparisons among three groups, one-way analysis of variance (ANOVA) was applied, followed by Tukey’s multiple-comparisons test. Statistical graphs were generated using GraphPad Prism 8.0. Differences were considered statistically significant at *p* < 0.05 (* *p* < 0.05, ** *p* < 0.01 and *** *p* < 0.001).

## 3. Results

### 3.1. Identification of Bovine Muscle Satellite Cells and Preadipocytes

**Figure 1:**
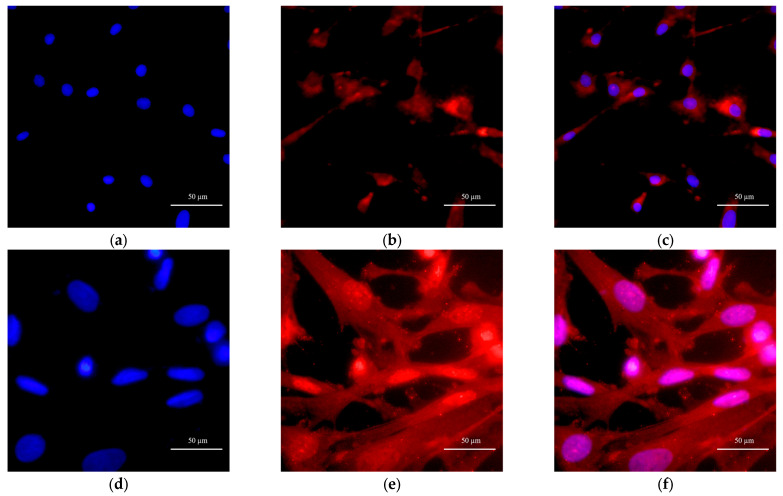
Identification of bovine muscle satellite cells and preadipocytes: (**a**–**c**) Identification of bovine preadipocytes by *PDGFRα* immunofluorescence (Scale bar = 50 µm); (**d**–**f**) Identification of bovine muscle satellite cells by *Pax7* immunofluorescence (Scale bar = 50 µm).

To clarify the origin and characteristics of the cell populations used in this study, bovine preadipocytes and muscle satellite cells were identified by immunofluorescence staining. Preadipocytes were stained for platelet-derived growth factor receptor α (PDGFRα), whereas muscle satellite cells were stained for paired box protein 7 (Pax7), with nuclei counterstained with DAPI. The results showed strong positive expression of the preadipocyte surface marker PDGFRα (Figure 1a–c), and muscle satellite cells specifically expressed the key nuclear transcription factor Pax7 (Figure 1d–f). PDGFRα is a well-recognized specific surface marker of preadipocytes, while Pax7 is a classical nuclear transcription factor expressed in both quiescent and activated muscle satellite cells. The positive signals of PDGFRα and Pax7 observed in this study confirm that the two primary cell populations cultured here are preadipocytes with proliferative and differentiative potential and muscle satellite cells with stem cell-like properties, providing a reliable cellular basis for the subsequent coculture and functional experiments.

### 3.2. Bovine Muscle Satellite Cells Inhibit Lipid Accumulation in Preadipocytes Under Co-Culture Conditions

**Figure 2:**
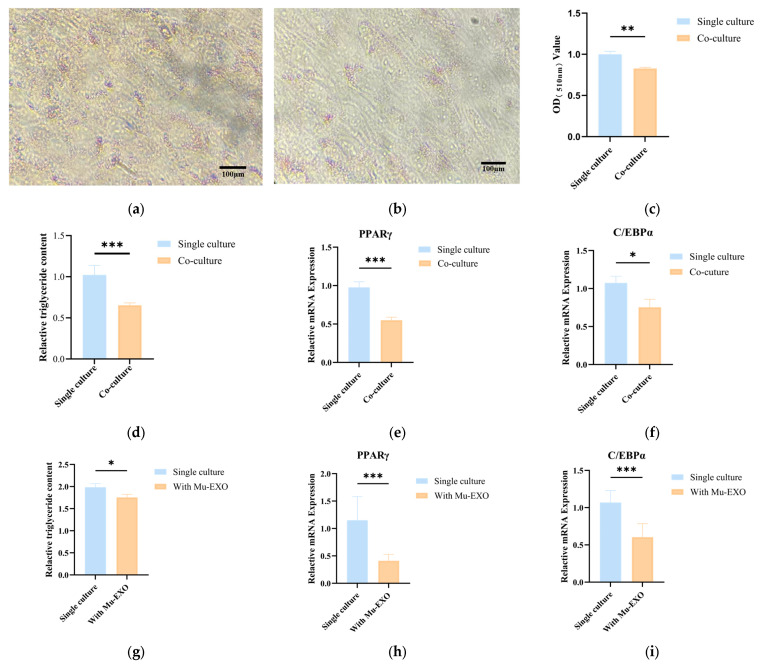
Effect of the Transwell co-culture system on lipid accumulation in preadipocytes: (**a**,**b**) Oil red o staining of adipocytes cultured alone and co-cultured with MuSCs; (**c**) Oil red O dye extraction; (**d**,**g**) Determination of TG content; (**e**,**h**), and (**f**,**i**), Relative mRNA expression of adipogenesis-related gene *PPARγ* and *C/EBPα*. The data are shown as mean ± SEM (*n* = 3 biological replicates). Statistical significance was assessed using independent two-sample *t*-test. * *p* < 0.05, ** *p* < 0.01 and *** *p* < 0.001.

To investigate whether bovine MuSCs affect adipogenic differentiation and lipid accumulation in preadipocytes, we established a Transwell co-culture system comprising bovine MuSCs and preadipocytes. The results showed that compared with adipocytes cultured alone, lipid droplet accumulation (*p* < 0.01) (Figure 2a–c) and TG content (*p* < 0.001) (Figure 2d) were significantly reduced in adipocytes co-cultured with MuSCs. Moreover, RT-qPCR analysis revealed that the mRNA expression of adipogenesis-related genes, *PPARγ* and CCAAT/enhancer-binding protein alpha (*C/EBPα*), in adipocytes was significantly downregulated after co-culture with MuSCs (*p* < 0.001, *p* < 0.05) (Figure 2e,f). To evaluate the effect of Mu-EXO on adipogenic differentiation, purified Mu-EXO were co-incubated with preadipocytes during induction. Relative to cells differentiated alone, Mu-EXO treated cultures exhibited a significant reduction in intracellular TG content (*p* < 0.05) (Figure 2g). Consistently, RT-qPCR revealed marked downregulation of the adipogenic regulators *PPARγ* and *C/EBPα* at the mRNA level in Mu-EXO treated adipocytes, indicating that Mu-EXO attenuate adipogenic differentiation (*p* < 0.001) (Figure 2h,i). These results indicate that bovine MuSCs inhibit adipogenic differentiation and lipid accumulation in adipocytes.

### 3.3. Isolation and Identification of Mu-EXO, Ad-EXO, and Co-EXO

**Figure 3:**
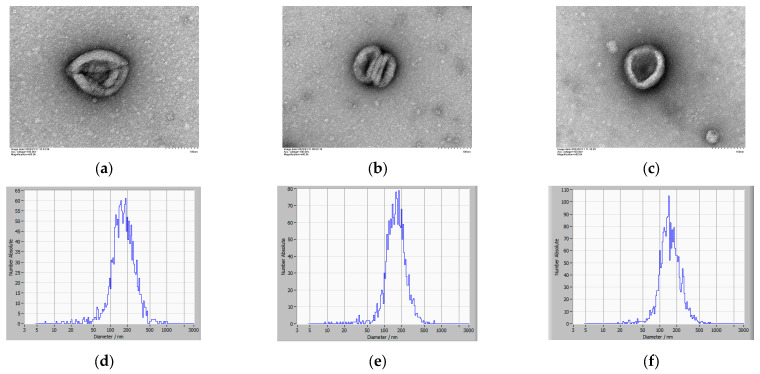
Isolation and Identification of Mu-EXO Ad-EXO, and Co-EXO: (**a**–**c**) Electron microscopic examination results of Mu-EXO Ad-EXO, and Co-EXO; (**d**–**f**) Detection results of particle size of Mu-EXO Ad-EXO, and Co-EXO. (*n* = 3 biological replicates).

To conduct morphological and particle size analyses of exosomes, Mu-EXO Ad-EXO, and Co-EXO were isolated and purified from cell culture supernatants. Under 60,000× magnification using TEM, exosomes exhibited a typical cup-shaped morphology with well-defined membrane boundaries (Figure 3a–c). NTA results further showed that the average particle sizes of Mu-EXO, Ad-EXO, and Co-EXO were 185.03 nm, 179.50 nm, and 164.87 nm, respectively (Figure 3d–f).

### 3.4. Small RNA-Seq Analysis of Mu-EXO, Ad-EXO, and Co-EXO

#### 3.4.1. Exosomal Small RNA Sequencing and Quality Control

To further explore the potential role of exosomal miRNAs in intercellular communication, Small RNA sequencing was performed on Mu-EXO, Ad-EXO, and Co-EXO, with three biological replicates per group. Raw reads were subjected to quality control using FastQC, and low-quality reads were removed to obtain high-quality clean reads suitable for downstream analysis. Detailed quality control results are presented in Table S5. Except for the sample Ad01-R2, all groups showed clean read Q20 values above 95% and Q30 values above 90%. These data demonstrate that the sequencing results were of high quality and reliable for subsequent analysis.

**Figure 4:**
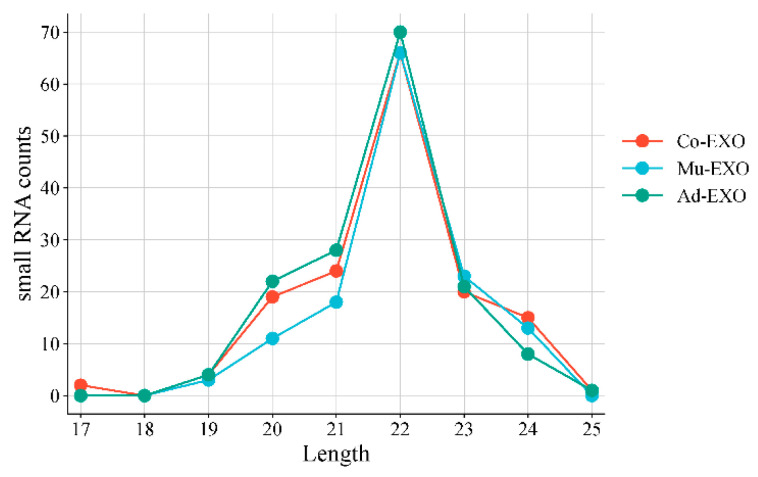
Length Distribution of Small RNA. (*n* = 3 biological replicates).

A statistical analysis of the length distribution of expressed miRNAs (reads > 0) was also performed. The results showed that the predominant peak in all samples was 22 nt. Only a small number of miRNAs were shorter than 20 nt or l longer than 24 nt, and the remaining miRNAs having lengths between 20 and 24 nt, whereas the majority ranged between 20 and 24 nt, consistent with known miRNA length features (Figure 4).

#### 3.4.2. Differential miRNA Analysis

**Figure 5:**
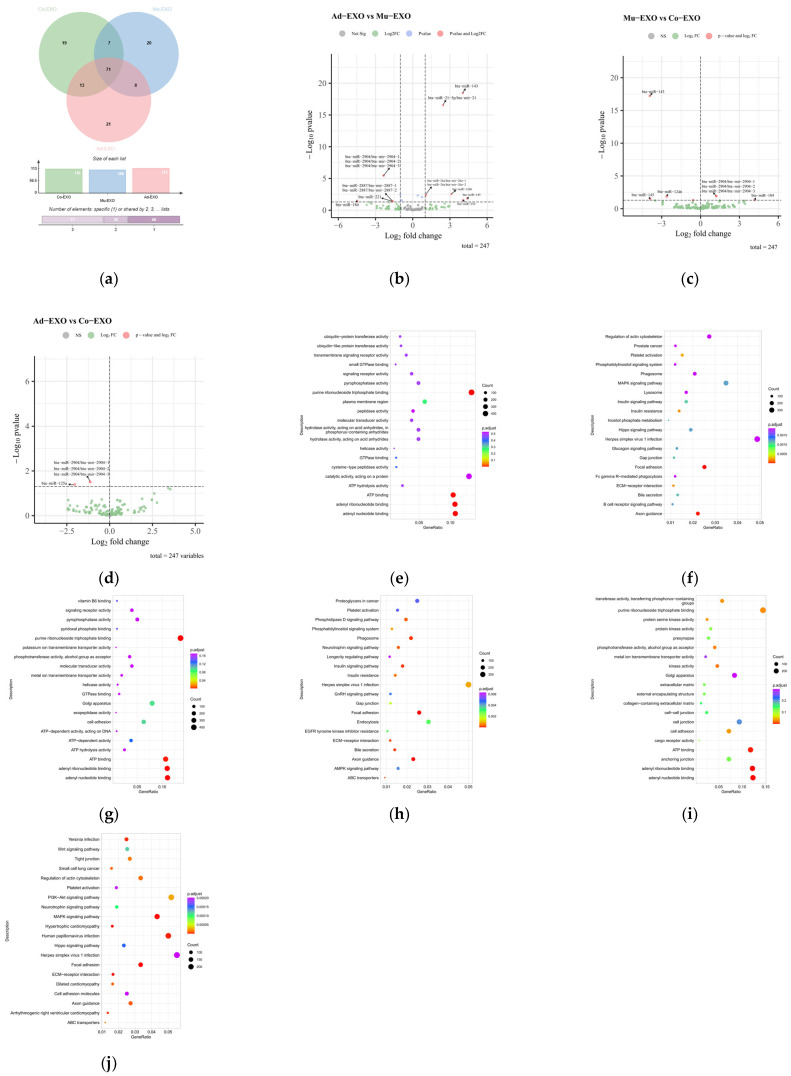
Expression profiling and differential analysis of exosomal miRNAs. (**a**) Venn diagram showing shared and group-specific expressed miRNAs among Mu-EXO, Ad-EXO, and Co-EXO. (**b**–**d**) Volcano plots of differentially expressed miRNAs for Ad-EXO vs. Mu-EXO, Mu-EXO vs. Co-EXO, and Ad-EXO vs. Co-EXO comparisons, respectively. (**e**–**j**) GO and KEGG enrichment analyses of predicted target genes of differentially expressed miRNAs in the corresponding comparisons (*n* = 3 biological replicates).

A total of 247 miRNAs were detected across all exosome samples. Among them, 71 miRNAs were commonly expressed in Mu-EXO, Ad-EXO, and Co-EXO; 20 miRNAs were specifically expressed in Mu-EXO; 21 miRNAs were specifically expressed in Ad-EXO; and 19 miRNAs were specifically expressed in Co-EXO (Figure 5a). The top 10 most abundant miRNAs in Mu-EXO were: bta-let-7a-5p, bta-let-7i, bta-let-7b, bta-let-7f, bta-miR-16a, bta-miR-2904, bta-miR-24-3p, bta-let-7e, bta-miR-29a, bta-miR-34a. The top 10 most highly expressed miRNAs in Ad-EXO were: bta-let-7i, bta-let-7a-5p, bta-miR-21-5p, bta-let-7b, bta-let-7f, bta-miR-143, bta-miR-29a, bta-miR-199a-3p, bta-miR-199c, bta-miR-26a. The top 10 most highly expressed miRNAs in Co-EXO were: bta-let-7i, bta-let-7a-5p, bta-let-7b, bta-miR-21-5p, bta-miR-143, bta-let-7f, bta-miR-16a, bta-miR-29a, bta-let-7g, bta-miR-24-3p. To prioritize candidate miRNAs from the sequencing screen, miRNAs meeting the thresholds of |log_2_ fold change| ≥ 1 and nominal *p* < 0.05 were selected. Under these screening criteria, 14 candidate miRNAs exhibited differential expression in the Ad-EXO vs. Mu-EXO comparison (7 upregulated and 7 downregulated; Figure 5b), 8 candidate miRNAs displayed differential expression in the Mu-EXO vs. Co-EXO comparison (4 upregulated and 4 downregulated; Figure 5c), and 4 candidate miRNAs were differentially expressed in the Ad-EXO vs. Co-EXO comparison, all of which were downregulated (Figure 5d). Across the three pairwise comparisons, several miRNAs showed nominal evidence of differential expression (*p* < 0.05). After Benjamini–Hochberg correction, two groups reached the conventional FDR threshold (padj < 0.05). Accordingly, the small RNA-seq results were used to prioritize candidate miRNAs, and the key candidates were subsequently evaluated by RT-qPCR and functional assays.

To obtain functional insights, GO and KEGG pathway enrichment analyses were performed on the predicted target genes of the candidate miRNAs from each comparison. The results indicated that these target genes participate in diverse processes, including molecular function, cellular components, biological processes, and biochemical metabolic pathways. In the Ad-EXO vs. Mu-EXO group, three KEGG pathways and two GO terms were associated with lipid metabolism: phosphatidylinositol signaling pathway, MAPK signaling pathway, insulin signaling pathway, ATP binding, and purine nucleoside triphosphate binding (Figure 5e,f). In the Mu-EXO vs. Co-EXO group, three KEGG pathways and two GO terms were enriched, including the insulin signaling pathway, AMPK signaling pathway, phosphatidylinositol signaling pathway, ATP binding, and purine nucleoside triphosphate binding (Figure 5g,h). In the Ad-EXO vs. Co-EXO group, three KEGG pathways and one GO term were related to lipid metabolism: Wnt signaling pathway, PI3K-Akt signaling pathway, MAPK signaling pathway, and ATP binding (Figure 5i,j).

Based on its abundance in Mu-EXO and its prioritization in the sequencing screen, bta-miR-2904 was selected as a representative candidate for subsequent RT-qPCR confirmation and functional assays.

### 3.5. Screening of Differentially Expressed miRNAs in Muscle-Derived Exosomes

**Figure 6:**
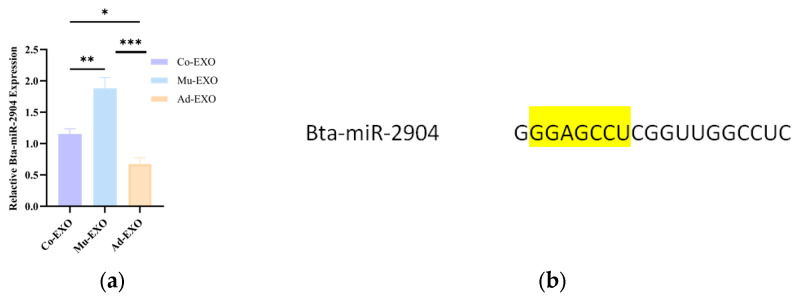
Relative expression and sequence features of bta-miR-2904. (**a**) Relative expression of bta-miR-2904 in Mu-EXO, Ad-EXO, and Co-EXO measured by RT-qPCR; (**b**) Bta-miR-2904 sequence (The seed regions indicated in yellow). The data are shown as mean ± SEM (*n* = 3 biological replicates). Statistical significance was assessed using one-way ANOVA followed by Tukey’s multiple-comparisons test. * *p* < 0.05, ** *p* < 0.01 and *** *p* < 0.001.

To prioritize candidate miRNAs potentially associated with adipogenesis, we first focused on downregulated miRNAs from the differentially expressed miRNAs in the Ad-EXO vs. Mu-EXO group, obtaining bta-miR-2904, bta-miR-2887, bta-miR-221, and bta-miR-184, all of which showed higher abundance in Mu-EXO than in Ad-EXO. Similarly, from the Ad-EXO vs. Co-EXO comparison, downregulated candidates miRNAs including bta-miR-2904 and bta-miR-125a, which were expressed higher in Co-EXO than in Ad-EXO. Based on the overlap of candidates from the two screening comparisons and the RT-qPCR results (Figure 6a), bta-miR-2904 was ultimately selected as the candidate miRNA for further analysis. Subsequently, bioinformatics analysis of bta-miR-2904 were performed using miRBase, TargetScan, and NCBI (Figure 6b). The results showed that the precursors bta-mir-2904-1, bta-mir-2904-2, and bta-mir-2904-3 were located on chromosomes 2 and 3 of cattle (*Bos taurus*). The mature bta-miR-2904 sequence was 19 nt in length. According to conservation score in Target Scan, bta-miR-2904 had a score of −1, indicating that it is not conserved among different species. This suggests that bta-miR-2904 is a bovine-specific miRNA.

### 3.6. Bta-miR-2904 Inhibits Adipogenic Differentiation of Preadipocytes

**Figure 7:**
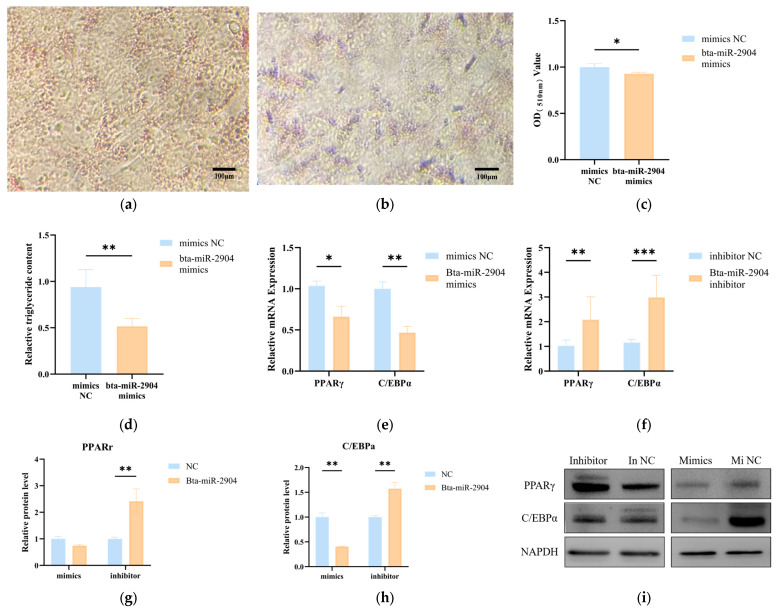
Effect of bta-miR-2904 on adipogenic differentiation of preadipocytes: (**a**,**b**) Oil red o staining after transfection of NC and mimics; (**c**) Oil red O dye extraction; (**d**) Determination of TG content; (**e**,**f**) Relative mRNA expression of adipogenesis-related gene *PPARγ* and *C/EBPα*; (**g**–**i**) Relative protein expression of adipogenesis-related gene PPARγ and C/EBPα. The data are shown as mean ± SEM (*n* = 3 biological replicates). Statistical significance was assessed using independent two-sample *t*-test. * *p* < 0.05, ** *p* < 0.01 and *** *p* < 0.001.

To further explore the regulatory role of bta-miR-2904 in preadipocyte adipogenic differentiation, we transfected bovine preadipocytes with bta-miR-2904 mimics and negative controls (NC). On day 9 of differentiation induction, cells were collected for Oil Red O staining and TG content determination. The results showed that compared with control group, transfection with bta-miR-2904 mimics significantly reduced lipid droplet accumulation (*p* < 0.05) (Figure 7a–c) and TG content in preadipocytes (*p* < 0.01) (Figure 7d). To further clarify the effect of bta-miR-2904 on adipogenesis-related genes in preadipocytes, we assessed the mRNA and protein expression levels of *PPARγ* and *C/EBPα*. Preadipocytes were transfected with bta-miR-2904 mimics, mimics NC, bta-miR-2904 inhibitor, or inhibitor NC, and total RNA and protein were extracted on day 6 of differentiation for RT-qPCR and Western blot analysis. RT-qPCR results revealed that transfection with bta-miR-2904 mimics significantly decreased *PPARγ* mRNA expression (*p* < 0.05) and markedly decreased *C/EBPα* mRNA expression (*p* < 0.01). Conversely, transfection with bta-miR-2904 inhibitors led to a significant increase in both *PPARγ* and *C/EBPα* mRNA expression (*p* < 0.01) (Figure 7e,f). Western blot results were consistent with RT-qPCR findings: after transfection with bta-miR-2904 mimics, the protein expression of PPARγ was decreased, and C/EBPα protein expression was markedly decreased (*p* < 0.01). After transfection with bta-miR-2904 inhibitor, whereas PPARγ and C/EBPα protein expression was significantly increased (*p* < 0.01) (Figure 7g–i). Collectively, these findings indicate that bta-miR-2904 functions as a negative regulator of adipogenic differentiation in bovine preadipocytes.

## 4. Discussion

IMF is a key factor affecting meat quality, and exosome-mediated crosstalk between skeletal muscle and adipose tissue plays a critical role in its regulation. Both preadipocytes and muscle cells originate from the mesoderm, and their proliferation and differentiation processes are under complex regulation by autocrine and paracrine signaling [27]. PDGFRα, a member of the receptor tyrosine kinase family, is predominantly expressed in mesenchymal/stromal progenitor cells in various tissues and is widely recognized as an important marker of the adipocyte lineage [28]. Pax7, a member of the paired box (Pax) transcription factor family, is regarded as the “gold-standard” marker of MuSCs in skeletal muscle—almost all satellite cells, whether quiescent or activated, express Pax7, which is crucial for determining their cell fate, maintaining stemness, and supporting muscle regeneration [29]. In our previous work, bovine MuSCs and preadipocytes were successfully isolated using the explant adherent culture method and enzymatic digestion, respectively, and systematically characterized by morphological assessment, immunofluorescence staining, and marker gene expression [20]. On this basis, the present study further verified these two cell populations by immunofluorescence. The results showed that Pax7 was positively expressed in MuSCs, with fluorescent signals mainly localized in the nuclei, whereas PDGFRα was positively expressed in preadipocytes, with signals predominantly distributed along the cell membrane, which is consistent with previous reports [30,31,32]. These findings, in conjunction with our earlier data, definitively confirm that the isolated cells are MuSCs possessing stem cell-like properties and preadipocytes at a progenitor stage with inherent adipogenic potential. This comprehensive characterization provides a robust and reliable cellular basis for the subsequent co-culture system and functional experiments.

Traditional studies have typically examined single cultures of myoblasts or preadipocytes. However, considering their common embryonic origin and close anatomical relationship in vivo, a muscle–adipose co-culture system provides a more physiologically relevant model to investigate the biological mechanisms underlying cell proliferation and differentiation. Based on our previous research of an adipose-muscle co-culture system, bovine muscle satellite cells and preadipocytes were co-cultured at a 2:1 ratio. Previous studies have demonstrated that in co-culture systems, C2C12 cells suppress the proliferation, differentiation, and lipid accumulation of 3T3-L1 cells [33,34]. Similarly, skeletal MuSCs from Tan sheep and pigs significantly inhibited the adipogenic capacity of preadipocytes under co-culture conditions [16,35,36]. Consistent with these findings, our results confirmed that bovine MuSCs significantly inhibited lipid accumulation and adipogenic differentiation of preadipocytes. Specifically, compared to monocultured controls, both co-culture with MuSCs and co-incubation with Mu-EXO significantly suppressed lipid droplet accumulation and TG content. At the molecular level, both mRNA levels of adipogenesis-related genes *PPARγ* and *C/EBPα* were downregulated to varying degrees.

As the largest core organ in the body, skeletal muscle not only regulates systemic metabolism and energy storage but also functions as an important endocrine organ. It secretes and releases a variety of bioactive molecules, including cytokines and exosomes, which mediate intercellular communication and maintain homeostasis [37]. Exosomes are nano-sized vesicles secreted by cells that function as intercellular messengers through the delivery of miRNAs, proteins, and lipids, thereby regulating cell development and differentiation. Compared to the vulnerability of free RNA to degradation by extracellular nucleases, the lipid bilayer membrane structure of exosomes protects their encapsulated RNA from enzymatic degradation, thereby markedly enhancing their extracellular stability. In this study, we successfully isolated and identified muscle-derived exosomes (Mu-EXO), adipose-derived exosomes (Ad-EXO), and co-culture exosomes (Co-EXO). High-throughput small RNA sequencing revealed distinct miRNA expression profiles among these exosomes. The identified miRNAs were predominantly 20–24 nt in length, consistent with known characteristics of mature miRNAs. Previous studies have identified differentially expressed miRNAs between muscle and adipose tissues, suggesting their significant association with adipogenesis and potential roles as candidate regulators of IMF deposition in cattle [38,39]. Interestingly, several of these differentially expressed miRNAs, which are significantly associated with fat deposition, overlap with the miRNAs identified in our study. This further validates the key role of muscle-derived exosomal miRNAs in regulating the muscle-adipose crosstalk. Functional enrichment analysis of the predicted target genes of differentially expressed miRNA demonstrated that many were involved in lipid metabolism-related pathways, including the Wnt signaling pathway, insulin signaling pathway, and ATP binding, suggesting that their targeted miRNAs may also participate in these processes to regulate the adipogenic differentiation of preadipocytes. Notably, bta-miR-2904, a bovine-specific miRNA, was significantly more abundant in Mu-EXO and Co-EXO than in Ad-EXO. This finding suggests that bta-miR-2904 may contribute to muscle-fat crosstalk, particularly in the regulation of adipogenesis. Previous studies have underscored the role of skeletal muscle-derived exosomes and their miRNAs in regulating adipocyte proliferation, differentiation, and lipid accumulation. For instance, skeletal muscle-derived exosomal miR-146a-5p has been shown to facilitate fatty acid uptake and adipogenesis by targeting Growth differentiation factor 5 (*GDF5*) and inhibiting the PPARγ signaling pathway to suppress preadipocyte differentiation and lipid accumulation [40].

In recent years, miRNAs have become a major research hotspot, and their roles in regulating adipogenic differentiation have been increasingly investigated. miR-143 was the first miRNA identified to promote adipocyte differentiation; it enhances adipogenesis by targeting mitogen-activated protein kinase 5 (*MAP2K5*) and functions as an early activator of adipocyte differentiation [41]. In contrast, miR-27a and miR-27b directly suppress *PPARγ* expression, thereby inhibiting adipogenic differentiation and attenuating lipid accumulation [42,43]. Similarly, miR-540 targets *PPARγ* to inhibit adipogenic differentiation of adipose-derived stem cells [44]. Collectively, these studies underscore the pivotal role of miRNAs in cellular differentiation and metabolism. They regulate key steps in adipocyte differentiation, lipolysis, and lipogenesis. Bioinformatics analysis of the differentially expressed bta-miR-2904 that we finally identified showed that its length is 19 nt, consistent with typical miRNA characteristics. According to the traditional definition, nucleotides 2–8 of an miRNA constitute the seed sequence, which is usually conserved in mammals. However, according to Target Scan, bta-miR-2904 has a conservation score of -1, indicating that it is not conserved across species. Since current evidence suggests that this miRNA is present only in cattle among mammals, it is considered a bovine-specific miRNA [45,46,47,48].

In Mu-EXO, miRNAs that are highly expressed may act as key regulators of fat deposition. Members of the let-7 family can regulate adipogenesis and lipid metabolism-related signaling pathways through competing endogenous RNA (ceRNA) networks [39,49,50,51]. Additionally, miR-16a [52], miR-34a [52], miR-24-3p [53], and miR-29a [54] regulate lipid homeostasis by participating in the proliferation and differentiation of adipocytes. Previous studies have demonstrated that bta-miR-2904 suppresses bovine viral diarrhea virus (BVDV) replication by targeting the autophagy-related gene Autophagy-related 13 (*ATG13*), thereby blocking virus-induced autophagy [45]. Additionally, this miRNA is abundant in bovine milk exosomes and exhibits exceptionally high isomiR diversity that suggests an ability to regulate a large number of target genes [55]. Moreover, bta-miR-2904 is also present in oviductal extracellular vesicles, which implies a potential involvement in embryo–maternal communication [56]. Although no studies have yet reported the role of miR-2904 in lipid metabolism, our small RNA-seq results identified bta-miR-2904 as a muscle-derived exosomal miRNA, suggesting its potential involvement in the adipogenic differentiation of preadipocytes. Our functional experiments further validated this hypothesis: Transfection with bta-miR-2904 mimics markedly reduced lipid droplet accumulation and TG content in bovine preadipocytes. In addition, bta-miR-2904 mimics also significantly downregulated the mRNA and protein expression levels of adipogenesis-related genes *PPARγ* and *C/EBPα*. Conversely, transfection with the bta-miR-2904 inhibitor produced the opposite effect, leading to increased lipid accumulation and upregulation of adipogenic differentiation-related genes. Together, these results provide strong evidence that bta-miR-2904 acts as a negative regulator of adipogenic differentiation in bovine preadipocytes and further suggest that the reduced adipogenic differentiation capacity of preadipocytes co-cultured with muscle satellite cells may be mediated, at least in part, by bta-miR-2904.

Despite the significant findings obtained in this study, several limitations warrant consideration. First, our study primarily employed an in vitro culture system. Although this approach is useful for dissecting specific intercellular interactions, it cannot fully reflect the complex physiological environment and systemic factors present in vivo. Therefore, future studies should validate these findings in appropriate in vivo models. Second, although bta-miR-2904 was confirmed as a key regulatory factor and its effects were supported by gain- and loss-of-function experiments, direct target validation was not performed in this study. In addition, other bioactive molecules within exosomes and their full regulatory mechanisms remain to be clarified. Subsequent investigations should focus on exploring the upstream regulators of bta-miR-2904 expression as well as its downstream targets to delineate the complete regulatory cascade. Third, the biological sample size in this study was relatively limited, and larger-scale studies will help to further evaluate inter-individual variability and strengthen the generalizability of our conclusions. Collectively, our results provide preliminary molecular insights into muscle–adipose tissue communication and offer a foundational reference for subsequent mechanistic studies and validation efforts aimed at regulating intramuscular fat deposition in beef cattle.

## 5. Conclusions

Our results show that, in the in vitro co-culture system established in this study, bovine muscle satellite cells significantly inhibited adipogenic differentiation of preadipocytes, as indicated by reduced lipid droplet accumulation and lower triglyceride content. Exosomal profiling further revealed that muscle-derived exosomes contained a distinct set of miRNAs, among which bta-miR-2904 was prioritized as a key candidate. Functional assays subsequently demonstrated that bta-miR-2904 reduced lipid accumulation and modulated the expression of adipogenesis-related genes, thereby exerting a negative regulatory effect on adipocyte differentiation. Collectively, these findings provide preliminary molecular insights into muscle-adipose tissue communication and offer a promising avenue for future studies exploring bta-miR-2904 as a molecular entry point for regulating fat deposition.

## Data Availability

MicroRNA sequencing data have been deposited in the CNCB GSA BioProject database under accession numbers PRJCA054990. The original contributions presented in this study are included in the article. Further inquiries can be directed to the corresponding authors.

## Supplementary Materials

The following supporting information can be downloaded at: https://www.mdpi.com/article/10.3390/ani16020218/s1, Table S1: TG content determination procedure; Table S2: Reverse transcription reaction system; Table S3: Reaction system of qRT-PCR; Table S4: Primer sequences used for qRT-PCR; Table S5: Table of data output quality; Table S6: Complete differential expression results for exosomal miRNAs across pairwise comparisons.

## Abbreviations

The following abbreviations are used in this manuscript:

IMF	Intramuscular fat
Mu-EXO	muscle-derived exosomes
Ad-EXO	adipose-derived exosomes
Co-EXO	co-culture exosomes
FAPs	fibro/adipogenic progenitors
MuSCs	skeletal muscle stem cells
S1pr3	Sphingosine-1-phosphate receptor 3
PPARγ	Peroxisome proliferator-activated receptor gamma
C/EBPα	CCAAT/enhancer-binding protein alpha
Runx1	Runt-related transcription factor 1
ATGL	Adipose triglyceride lipase
HSL	Hormone-sensitive lipase
PLIN1	Perilipin 1
MSTN	myostatin
Smad3	SMAD family member 3
MVBs	multivesicular bodies
EVs	extracellular vesicles
UCP1	uncoupling protein 1
MAP2K5	mitogen-activated protein kinase 5
ATG13	Autophagy-related 13
GDF5	Growth differentiation factor 5
TEM	Transmission Electron Microscopy
NTA	Nanoparticle Tracking Analysis
ceRNA	competing endogenous RNA

## References

[1] Schumacher M., DelCurto-Wyffels H., Thomson J., Boles J. (2022). Fat Deposition and Fat Effects on Meat Quality—A Review. Animals.

[2] Tan Z., Jiang H. (2024). Molecular and Cellular Mechanisms of Intramuscular Fat Development and Growth in Cattle. Int. J. Mol. Sci..

[3] Park S.J., Beak S.-H., Jung D.J.S., Kim S.Y., Jeong I.H., Piao M.Y., Kang H.J., Fassah D.M., Na S.W., Yoo S.P. (2018). Genetic, Management, and Nutritional Factors Affecting Intramuscular Fat Deposition in Beef Cattle—A Review. Asian-Australas. J. Anim. Sci..

[4] Lee M.-J., Wu Y., Fried S.K. (2013). Adipose Tissue Heterogeneity: Implication of Depot Differences in Adipose Tissue for Obesity Complications. Mol. Asp. Med..

[5] Yu Y., Su Y., Wang G., Lan M., Liu J., Garcia Martin R., Brandao B.B., Lino M., Li L., Liu C. (2024). Reciprocal Communication between FAPs and Muscle Cells via Distinct Extracellular Vesicle MiRNAs in Muscle Regeneration. Proc. Natl. Acad. Sci. USA.

[6] Vliora M., Grillo E., Corsini M., Ravelli C., Nintou E., Karligiotou E., Flouris A.D., Mitola S. (2022). Irisin Regulates Thermogenesis and Lipolysis in 3T3-L1 Adipocytes. Biochim. Biophys. Acta (BBA)-Gen. Subjects.

[7] Liu K., Zhang X., Wei W., Liu X., Tian Y., Han H., Zhang L., Wu W., Chen J. (2019). Myostatin/SMAD4 Signaling-Mediated Regulation of MiR-124-3p Represses Glucocorticoid Receptor Expression and Inhibits Adipocyte Differentiation. Am. J. Physiol.-Endocrinol. Metab..

[8] Ren H., Xiao W., Qin X., Cai G., Chen H., Hua Z., Cheng C., Li X., Hua W., Xiao H. (2020). Myostatin Regulates Fatty Acid Desaturation and Fat Deposition through MEF2C/MiR222/SCD5 Cascade in Pigs. Commun. Biol..

[9] Guo L., Xu J., Zhou W., Chen S., Shi H., Han M., Yang Z., Duan Y., Pang W., Yin Y. (2025). Metabolome and RNA-Seq Reveal Discrepant Metabolism and Secretory Metabolism Profile in Skeletal Muscle between Obese and Lean Pigs at Different Ages. Sci. China Life Sci..

[10] Ferguson S.W., Nguyen J. (2016). Exosomes as Therapeutics: The Implications of Molecular Composition and Exosomal Heterogeneity. J. Control. Release.

[11] Friedman R.C., Farh K.K.-H., Burge C.B., Bartel D.P. (2009). Most Mammalian MRNAs Are Conserved Targets of MicroRNAs. Genome Res..

[12] Krylova S.V., Feng D. (2023). The Machinery of Exosomes: Biogenesis, Release, and Uptake. Int. J. Mol. Sci..

[13] Deng Z., Poliakov A., Hardy R.W., Clements R., Liu C., Liu Y., Wang J., Xiang X., Zhang S., Zhuang X. (2009). Adipose Tissue Exosome-Like Vesicles Mediate Activation of Macrophage-Induced Insulin Resistance. Diabetes.

[14] Zhao R., Zhao T., He Z., Cai R., Pang W. (2021). Composition, Isolation, Identification and Function of Adipose Tissue-Derived Exosomes. Adipocyte.

[15] Li W., Wen S., Wu J., Zeng B., Chen T., Luo J., Shu G., Wang S., Zhang Y., Xi Q. (2021). Comparative Analysis of MicroRNA Expression Profiles Between Skeletal Muscle- and Adipose-Derived Exosomes in Pig. Front. Genet..

[16] Qin M., Xing L., Wen S., Luo J., Sun J., Chen T., Zhang Y., Xi Q. (2024). Heterogeneity of Extracellular Vesicles in Porcine Myoblasts Regulates Adipocyte Differentiation. Sci. Rep..

[17] Qiu M., Li T., Wang B., Gong H., Huang T. (2020). MiR-146a-5p Regulated Cell Proliferation and Apoptosis by Targeting SMAD3 and SMAD4. Protein Pept. Lett..

[18] Schjølberg T., Asoawe L., Krapf S., Rustan A., Thoresen G., Haugen F. (2022). Experimental Models for Cold Exposure of Muscle In Vitro and In Vivo. Bio-Protocol.

[19] Wang D., Zhang X., Li Y., Jia L., Zhai L., Wei W., Zhang L., Jiang H., Bai Y. (2022). Exercise-Induced Browning of White Adipose Tissue and Improving Skeletal Muscle Insulin Sensitivity in Obese/Non-Obese Growing Mice: Do Not Neglect Exosomal MiR-27a. Front. Nutr..

[20] Meng G., Zhang J., Wu Z., Song J., Sun Q., Zhang X., Sun M., Yi Y., Xia G. (2025). Bovine Adipocyte-Derived Exosomes Transport LncRNAs to Regulate Adipogenic Transdifferentiation of Bovine Muscle Satellite Cells. Animals.

[21] Guo L., Cui H., Zhao G., Liu R., Li Q., Zheng M., Guo Y., Wen J. (2018). Intramuscular Preadipocytes Impede Differentiation and Promote Lipid Deposition of Muscle Satellite Cells in Chickens. BMC Genom..

[22] Kuppusamy P., Kim D., Soundharrajan I., Hwang I., Choi K.C. (2020). Adipose and Muscle Cell Co-Culture System: A Novel In Vitro Tool to Mimic the In Vivo Cellular Environment. Biology.

[23] Sahraeian S.M.E., Mohiyuddin M., Sebra R., Tilgner H., Afshar P.T., Au K.F., Bani Asadi N., Gerstein M.B., Wong W.H., Snyder M.P. (2017). Gaining Comprehensive Biological Insight into the Transcriptome by Performing a Broad-Spectrum RNA-Seq Analysis. Nat. Commun..

[24] Love M.I., Huber W., Anders S. (2014). Moderated Estimation of Fold Change and Dispersion for RNA-Seq Data with DESeq2. Genome Biol..

[25] Huang D.W., Sherman B.T., Lempicki R.A. (2009). Bioinformatics Enrichment Tools: Paths toward the Comprehensive Functional Analysis of Large Gene Lists. Nucleic Acids Res..

[26] Kanehisa M. (2000). KEGG: Kyoto Encyclopedia of Genes and Genomes. Nucleic Acids Res..

[27] Kokta T.A., Dodson M.V., Gertler A., Hill R.A. (2004). Intercellular Signaling between Adipose Tissue and Muscle Tissue. Domest. Anim. Endocrinol..

[28] Giuliani G., Rosina M., Reggio A. (2022). Signaling Pathways Regulating the Fate of Fibro/Adipogenic Progenitors (FAPs) in Skeletal Muscle Regeneration and Disease. FEBS J..

[29] Zammit P.S., Relaix F., Nagata Y., Ruiz A.P., Collins C.A., Partridge T.A., Beauchamp J.R. (2006). Pax7 and Myogenic Progression in Skeletal Muscle Satellite Cells. J. Cell Sci..

[30] Reggio A., Rosina M., Palma A., Cerquone Perpetuini A., Petrilli L.L., Gargioli C., Fuoco C., Micarelli E., Giuliani G., Cerretani M. (2020). Adipogenesis of Skeletal Muscle Fibro/Adipogenic Progenitors Is Affected by the WNT5a/GSK3/β-Catenin Axis. Cell Death Differ..

[31] Flores-Opazo M., Kopinke D., Helmbacher F., Fernández-Verdejo R., Tuñón-Suárez M., Lynch G.S., Contreras O. (2024). Fibro-Adipogenic Progenitors in Physiological Adipogenesis and Intermuscular Adipose Tissue Remodeling. Mol. Asp. Med..

[32] Zhao T., Tian T., Yu H., Cao C., Zhang Z., He Z., Ma Z., Cai R., Li F., Pang W. (2024). Identification of Porcine Fast/Slow Myogenic Exosomes and Their Regulatory Effects on Lipid Accumulation in Intramuscular Adipocytes. J. Anim. Sci. Biotechnol..

[33] Chu W., Wei W., Yu S., Han H., Shi X., Sun W., Gao Y., Zhang L., Chen J. (2016). C2C12 Myotubes Inhibit the Proliferation and Differentiation of 3T3-L1 Preadipocytes by Reducing the Expression of Glucocorticoid Receptor Gene. Biochem. Biophys. Res. Commun..

[34] Pandurangan M., Ravikumar S. (2014). Impact of Stress Hormone on Adipogenesis in the 3T3-L1 Adipocytes. Cytotechnology.

[35] Xu X., Zhao R., Ma W., Zhao Q., Zhang G. (2022). Comparison of Lipid Deposition of Intramuscular Preadipocytes in Tan Sheep Co-cultured with Satellite Cells or Alone. J. Anim. Physiol. Anim. Nutr..

[36] Hausman G.J., Poulos S.P. (2005). A Method to Establish Co-Cultures of Myotubes and Preadipocytes from Collagenase Digested Neonatal Pig Semitendinosus Muscles1. J. Anim. Sci..

[37] Soriano-Cruz M., Vázquez-González W.G., Molina-Vargas P., Faustino-Trejo A., Chávez-Rueda A.K., Legorreta-Haquet M.V., Aguilar-Ruíz S.R., Chávez-Sánchez L. (2024). Exosomes as Regulators of Macrophages in Cardiovascular Diseases. Biomedicines.

[38] Huang J., Wang S., Feng X., Liu X., Zhao J., Zheng Q., Wei X., Ma Y. (2019). MiRNA Transcriptome Comparison between Muscle and Adipose Tissues Indicates Potential MiRNAs Associated with Intramuscular Fat in Chinese Swamp Buffalo. Genome.

[39] Qiu J., Ma Z., Hong Z., Yin X., Chen Y., Ahmed H.Q., Zan L., Li A. (2025). Comparative Analysis of the Whole Transcriptome Landscapes of Muscle and Adipose Tissue in Qinchuan Beef Cattle. BMC Genom..

[40] Qin M., Xing L., Wu J., Wen S., Luo J., Chen T., Fan Y., Zhu J., Yang L., Liu J. (2023). Skeletal Muscle-Derived Exosomal MiR-146a-5p Inhibits Adipogenesis by Mediating Muscle-Fat Axis and Targeting GDF5-PPARγ Signaling. Int. J. Mol. Sci..

[41] Esau C., Kang X., Peralta E., Hanson E., Marcusson E.G., Ravichandran L.V., Sun Y., Koo S., Perera R.J., Jain R. (2004). MicroRNA-143 Regulates Adipocyte Differentiation. J. Biol. Chem..

[42] Kim S.Y., Kim A.Y., Lee H.W., Son Y.H., Lee G.Y., Lee J.-W., Lee Y.S., Kim J.B. (2010). MiR-27a Is a Negative Regulator of Adipocyte Differentiation via Suppressing PPARγ Expression. Biochem. Biophys. Res. Commun..

[43] Karbiener M., Fischer C., Nowitsch S., Opriessnig P., Papak C., Ailhaud G., Dani C., Amri E.-Z., Scheideler M. (2009). MicroRNA MiR-27b Impairs Human Adipocyte Differentiation and Targets PPARγ. Biochem. Biophys. Res. Commun..

[44] Chen L., Chen Y., Zhang S., Ye L., Cui J., Sun Q., Li K., Wu H., Liu L. (2015). MiR-540 as a Novel Adipogenic Inhibitor Impairs Adipogenesis Via Suppression of PPARγ. J. Cell. Biochem..

[45] Yang N., Hu N., Zhang J., Yi J., Wang Z., Wang Y., Wu P., Chen C. (2023). Bta-MiR-2904 Inhibits Bovine Viral Diarrhea Virus Replication by Targeting Viral-Infection-Induced Autophagy via ATG13. Arch. Virol..

[46] Colitti M., Sgorlon S., Licastro D., Stefanon B. (2019). Differential Expression of MiRNAs in Milk Exosomes of Cows Subjected to Group Relocation. Res. Vet. Sci..

[47] Turri F., Capra E., Lazzari B., Cremonesi P., Stella A., Pizzi F. (2021). A Combined Flow Cytometric Semen Analysis and MiRNA Profiling as a Tool to Discriminate Between High- and Low-Fertility Bulls. Front. Vet. Sci..

[48] Cui X., Zhang S., Zhang Q., Guo X., Wu C., Yao M., Sun D. (2020). Comprehensive MicroRNA Expression Profile of the Mammary Gland in Lactating Dairy Cows with Extremely Different Milk Protein and Fat Percentages. Front. Genet..

[49] Zhang Y.-Y., Wang H.-B., Wang Y.-N., Wang H.-C., Zhang S., Hong J.-Y., Guo H.-F., Chen D., Yang Y., Zan L.-S. (2017). Transcriptome Analysis of MRNA and MicroRNAs in Intramuscular Fat Tissues of Castrated and Intact Male Chinese Qinchuan Cattle. PLoS ONE.

[50] Gao Y., Wang S., Ma Y., Lei Z., Ma Y. (2022). Circular RNA Regulation of Fat Deposition and Muscle Development in Cattle. Vet. Med. Sci..

[51] Shao J., Jiang G., Li Y., Wang M., Tang T., Wang J., Jia X., Lai S. (2024). Let-7a-5p Regulates Animal Lipid Accumulation by Targeting Srebf2 and Thbs1 Signaling. Int. J. Mol. Sci..

[52] Ciecierska A., Gorji A.E., Majewska A., Sadkowski T. (2025). Expression of MiRNA in the Semitendinosus Muscle of Cattle Breeds with Varying Intramuscular Fat Deposition. Genes.

[53] Lin Z., Tang Y., Li Z., Li J., Yu C., Yang C., Liu L., Wang Y., Liu Y. (2022). MiR-24-3p Dominates the Proliferation and Differentiation of Chicken Intramuscular Preadipocytes by Blocking ANXA6 Expression. Genes.

[54] Zhao L., Zhou L., Hao X., Wang L., Han F., Liu L., Duan X., Guo F., He J., Liu N. (2021). Identification and Characterization of Circular RNAs in Association with the Deposition of Intramuscular Fat in Aohan Fine-Wool Sheep. Front. Genet..

[55] Sun J., Aswath K., Schroeder S.G., Lippolis J.D., Reinhardt T.A., Sonstegard T.S. (2015). MicroRNA Expression Profiles of Bovine Milk Exosomes in Response to Staphylococcus Aureus Infection. BMC Genom..

[56] Hamdi M., Sánchez J.M., Fernandez-Fuertes B., Câmara D.R., Bollwein H., Rizos D., Bauersachs S., Almiñana C. (2024). Oviductal Extracellular Vesicles MiRNA Cargo Varies in Response to Embryos and Their Quality. BMC Genom..

