# Supplementary material for: Bovine Muscle Satellite Cell-Derived Exosomes Modulate Preadipocyte Adipogenesis via bta-miR-2904
Source: Animals (Basel). 2026 Jan 12;16(2):218. doi: 10.3390/ani16020218 (PMC12837153; doi:10.3390/ani16020218)
Supplement: Supplementary file 1 [file animals-16-00218-s001.zip › animals-4063691-SI.pdf]

**Table S1** TG content determination procedure

| | Sample Tube | Calibration Tube | Blank Tube |
|---|---|---|---|
| Sample（μL） | 2.5 | - | - |
| Calibration（μL） | - | 2.5 | - |
| Distilled Water（μL） | - | - | 2.5 |
| Working Solution（μL） | 250 | 250 | 250 |

**Table S2** Reverse transcription reaction system

| Composition | System |
|---|---|
| 5× FastKing-RT SuperMix | 4 μL |
| Total RNA | 50 ng-2 μg |
| RNase-free water | To 20 μL |
| Total volume | 20 μL |

**Table S3** Reaction system of qRT-PCR

| Composition | Volume (μL) | Final concentration |
|---|---|---|
| SuperReal PreMix Plus (2×) | 5 | 1× |
| PCR Forward Primer (10 μM) | 0.3 | 0.3 μM |
| PCR Reverse Primer (10 μM) | 0.3 | 0.3 μM |
| cDNA | 1 | - |
| ROX Reference Dye (50×) | 0.2 | 1× |
| Double-distilled water (dd$H_2O$) | 3.2 | - |
| Total volume | 10 | |

**Table S4** Primer sequences used for qRT-PCR

| Genes | Accession Number | Primer Sequence (5'-3') | Product size (bp) |
|---|---|---|---|
| *PPARγ* | NM_181024.2 | F: ATCAAGTTCAAGCACACATCAG<br>R: GTTCAAGTCAAGGTTCACAA | 154 |
| *CEBP/α* | NM_176784.2 | F: ACAAGAACAGCAACGAGTA<br>R: GGTCATTGTCACTGGTCAG | 134 |
| *GAPDH* | NSO_4761240 | F: ACTCTGGCAAAGTGGATGTTGTC<br>R: GCATCACCCCACTTGATGTTG | 95 |

* F indicates the forward primer; R indicates the reverse primer.

**Table S5** Table of data output quality

| Group | Reads raw | Q20_ raw | Q30_ raw | raw_ GC | reads_ clean | Q20_ clean | Q30_ clean | clean_ GC |
|---|---|---|---|---|---|---|---|---|
| Co-EXO01_R1 | 12919313 | 94.18% | 88.21% | 58% | 6612704 | 98.63% | 95.83% | 53% |
| Co-EXO01_R2 | 12919313 | 94.85% | 90.22% | 55% | 12890228 | 96.50% | 92.57% | 49% |
| Co-EXO02_R1 | 11776748 | 94.98% | 89.54% | 58% | 6528873 | 98.71% | 95.98% | 53% |

| | | | | | | | | |
|---|---|---|---|---|---|---|---|---|
| Co-EXO02_R2 | 11776748 | 94.23% | 89.22% | 53% | 11745895 | 96.06% | 91.79% | 47% |
| Co-EXO03_R1 | 9831649 | 91.58% | 84.16% | 58% | 5304306 | 98.24% | 95.09% | 53% |
| Co-EXO03_R2 | 9831649 | 94.39% | 89.40% | 55% | 9798018 | 96.25% | 92.03% | 49% |
| Mu-EXO01_R1 | 13875736 | 88.09% | 81.20% | 58% | 8207065 | 98.39% | 95.48% | 53% |
| Mu-EXO01_R2 | 13875736 | 94.33% | 88.96% | 53% | 13840449 | 95.87% | 91.08% | 47% |
| Mu-EXO02_R1 | 13036214 | 93.58% | 87.23% | 57% | 7543355 | 98.60% | 95.82% | 52% |
| Mu-EXO02_R2 | 13036214 | 94.54% | 89.32% | 55% | 13001382 | 96.15% | 91.55% | 49% |
| Mu-EXO03_R1 | 14536889 | 93.31% | 86.99% | 58% | 8085271 | 98.63% | 95.84% | 53% |
| Mu-EXO03_R2 | 14536889 | 94.43% | 89.41% | 53% | 14504835 | 96.01% | 91.59% | 47% |
| Ad-EXO01_R1 | 12572379 | 86.07% | 79.47% | 60% | 6112428 | 98.11% | 94.80% | 54% |
| Ad-EXO01_R2 | 12572379 | 92.93% | 86.76% | 53% | 12508606 | 94.61% | 89.11% | 47% |
| Ad-EXO02_R1 | 11604130 | 92.91% | 85.99% | 56% | 7094087 | 98.34% | 94.95% | 54% |
| Ad-EXO02_R2 | 11604130 | 94.03% | 88.59% | 52% | 11568838 | 95.45% | 90.58% | 48% |
| Ad-EXO03_R1 | 10187714 | 95.18% | 89.88% | 57% | 5698231 | 98.69% | 96.01% | 53% |
| Ad-EXO03_R2 | 10187714 | 94.27% | 89.27% | 52% | 10163534 | 96.00% | 91.70% | 47% |

**Table S6** Complete differential expression results for exosomal miRNAs across pairwise comparisons.

| Comparison | miRNA_ID | log2FC | P_value | padj |
|---|---|---|---|---|
| Mu-EXO vs Ad-EXO | bta-miR-143 | 4.03586970235295 | 3.49046E-19 | 4.43288E-17 |
| Mu-EXO vs Ad-EXO | bta-miR-21-5p | 2.445217494 | 2.92663E-17 | 1.85841E-15 |
| Mu-EXO vs Ad-EXO | bta-miR-2904 | -2.347423729 | 3.47692E-06 | 8.83139E-05 |
| Mu-EXO vs Ad-EXO | bta-miR-2904 | -2.347423729 | 3.47692E-06 | 8.83139E-05 |
| Mu-EXO vs Ad-EXO | bta-miR-2904 | -2.347423729 | 3.47692E-06 | 8.83139E-05 |
| Mu-EXO vs Ad-EXO | bta-miR-1246 | 3.116563248 | 0.00304363 | 0.060432965 |
| Mu-EXO vs Ad-EXO | bta-miR-26a | 1.063010521 | 0.004470455 | 0.060432965 |
| Mu-EXO vs Ad-EXO | bta-miR-26a | 1.063010521 | 0.004470455 | 0.060432965 |
| Mu-EXO vs Ad-EXO | bta-miR-145 | 4.396132127 | 0.01494477 | 0.158165486 |
| Mu-EXO vs Ad-EXO | bta-miR-210 | 4.048889728 | 0.025064605 | 0.244861907 |
| Mu-EXO vs Ad-EXO | bta-miR-184 | -4.467923051 | 0.032923666 | 0.276579437 |
| Mu-EXO vs Ad-EXO | bta-miR-2887 | -1.628495597 | 0.037022444 | 0.276579437 |
| Mu-EXO vs Ad-EXO | bta-miR-2887 | -1.628495597 | 0.037022444 | 0.276579437 |
| Mu-EXO vs Ad-EXO | bta-miR-221 | -1.658578621 | 0.040806553 | 0.2879129 |
| Ad-EXO vs Co-EXO | bta-miR-2904 | -1.133468178 | 0.030807632 | 0.754081556 |
| Ad-EXO vs Co-EXO | bta-miR-2904 | -1.133468178 | 0.030807632 | 0.754081556 |
| Ad-EXO vs Co-EXO | bta-miR-2904 | -1.133468178 | 0.030807632 | 0.754081556 |
| Ad-EXO vs Co-EXO | bta-miR-125a | -2.03305943 | 0.040058734 | 0.98489355 |
| Mu-EXO vs Co-EXO | bta-miR-143 | -3.920085083 | 6.13E-18 | 1.51E-15 |

| | | | | |
|---|---|---|---|---|
| Mu-EXO vs Co-EXO | bta-miR-21-5p | -1.852085983 | 4.64E-10 | 5.73E-08 |
| Mu-EXO vs Co-EXO | bta-miR-2904 | 1.213955551 | 0.01194508 | 0.491739019 |
| Mu-EXO vs Co-EXO | bta-miR-2904 | 1.213955551 | 0.01194508 | 0.491739019 |
| Mu-EXO vs Co-EXO | bta-miR-2904 | 1.213955551 | 0.01194508 | 0.491739019 |
| Mu-EXO vs Co-EXO | bta-miR-1246 | -2.619185617 | 0.01458050 | 0.514483431 |
| Mu-EXO vs Co-EXO | bta-miR-145 | -3.88518082 | 0.03486322 | 0.72024935 |
| Mu-EXO vs Co-EXO | bta-miR-184 | 4.175424978 | 0.04621945 | 0.72024935 |
